# PReS-FINAL-2002: Adverse events during anti-TNF therapy in patients with JIA

**DOI:** 10.1186/1546-0096-11-S2-O5

**Published:** 2013-12-05

**Authors:** M Tarkiainen, P Tynjälä, P Vähäsalo, P Lahdenne

**Affiliations:** 1 Children's Hospital, Helsinki University Central Hospital, Finland; 2 University of Helsinki, Helsinki, Finland; 3 Oulu University Central Hospital, Oulu, Finland

## Introduction

Biologic agents for rheumatic diseases are powerful drugs which may cause adverse events (AEs). It is important to characterize AEs in long-term real-life setting in patients with JIA.

## Objectives

This study aimed to investigate the incidence of AEs during biologic therapy in patients with JIA.

## Methods

Data were collected from medical charts of 348 consecutive patients with JIA from three tertiary centres in Finland. Biologic therapy for these patients was started 1999-2009. AEs were categorized according to modified Common Terminology Criteria for Adverse Events 4.0(CTCAE) -criteriae.

## Results

The follow-up time of the patients was altogether 1516 patient-years (py): 710 for etanercept, 591 for infliximab, 188 for adalimumab, and 27 for other biologic drugs. 216 (62%) patients were female. Median age at onset of JIA was 4.7 years (0.7 to 15.9). Anti-TNF therapy was started at a median age of 10.8 years (2.2 to 19.2). All patients had either received DMARDs prior to anti-TNF or continued receiving them concomitantly. Median follow-up time per patient in this study was 50.5 months (1.0 to 154.7).

318 patients (91%) had at least one AE. Most common AEs were infections; such as upper respiratory tract infections, otitis media, sinusitis, and gastroenteritis [44.4, 6.6, 5.7, and 4.8 per 100 py].

121 (34.7%) patients had a serious AE (SAE). Forty-four patients had a serious infectious AE (3.8 AEs/100 py), of which septicaemia (0.9/100 py) and pneumonia (0.7/100 py) were the most common.

The frequency of alanine aminotransferase elevation >5 times upper limit of normal was 1.7 per 100 py, and of neutropenia 2.2 per 100 py.

AEs of special interest included new-onset colitis ulcerosa, Crohn's disease, psoriasis, alopecia, or vasculitis in 4, 2, 9, 6, or 2 patients, respectively.

No malignancies were observed. Three patients had each a cyst in spleen, brain or breast, and two had abnormal pap smear findings. Two patients with severe systemic onset JIA died after initiation of biologic treatment. Biologic drugs didn't seem to differ from each other in terms of the number of associated AEs. Since the patients may have switched between several biologic agents, comparisons between drug treatment groups were not possible.

## Conclusion

Anti-TNF therapy is relatively safe in hands of pediatric rheumatologists. However, one third of patients had a serious AE, and for 100 patient-years, 3.8 serious infections were observed. Thus, careful monitoring is needed when treating pediatric patients with anti-TNFs or other biologic agents.

## Disclosure of interest

None declared.

